# Sino-Orbital Aspergillosis in a Kidney Transplant Recipient

**DOI:** 10.1155/2022/5946446

**Published:** 2022-04-14

**Authors:** Christian Maalouli, Julien De Greef, Thierry Duprez, Arnaud Devresse, Caroline Huart, Maëlle Coutel, Nathalie Demoulin, Leïla Belkhir, Nada Kanaan

**Affiliations:** ^1^Division of Nephrology, Cliniques Universitaires Saint-Luc, Université Catholique de Louvain, Brussels, Belgium; ^2^Division of Internal Medicine and Infectious Diseases, Cliniques Universitaires Saint-Luc, Université Catholique de Louvain, Brussels, Belgium; ^3^Division of Medical Imaging and Radiology, Cliniques Universitaires Saint-Luc, Université Catholique de Louvain, Brussels, Belgium; ^4^Division of Otorhinolaryngology, Cliniques Universitaires Saint-Luc, Université Catholique de Louvain, Brussels, Belgium; ^5^Division of Ophthalmology, Cliniques Universitaires Saint-Luc, Université Catholique de Louvain, Brussels, Belgium

## Abstract

Sino-orbital aspergillosis is a rare and severe infection mostly seen in immunocompromised individuals in which diagnosis may be challenging with potentially life-threatening consequences. Infection usually starts in the paranasal sinuses with secondary spreading to the adjacent orbits. Here, we report the case of a kidney transplant recipient who presented with proven invasive sino-orbital aspergillosis resulting in irreversible loss of vision despite surgical management and antifungal therapy. We review the literature with a focus on clinical presentation, diagnostic tools, and recommended treatment in the context of kidney transplantation.

## 1. Introduction

Orbital aspergillosis is a rare orbital infection, mainly seen in immunocompromised individuals. It usually occurs as a complication of adjacent sphenoid or ethmoid sinusitis. Because of unspecific symptoms, diagnosis can be delayed with life-threatening consequences [1, 2]. Here, we describe a kidney transplant recipient (KTR) who suffered from a localized invasive sino-orbital aspergillosis with irreversible vision loss.

## 2. Case Presentation

**Figure 1:**
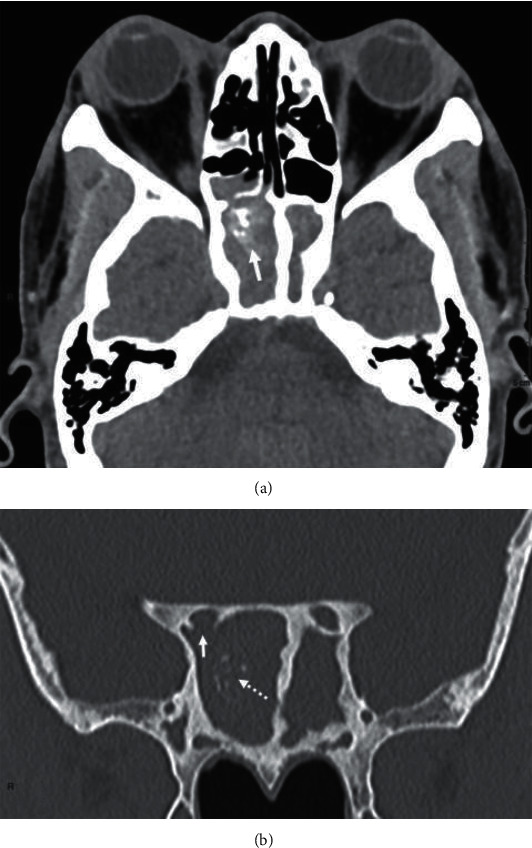
Head computed tomography scan. (a) Unenhanced axial transverse view in parenchymal reconstruction algorithm showing filling of the sphenoid sinus and of the right-sided posterior ethmoid cell by inflammatory material. Observe heterogeneous and amorphous calcium deposits within the right sphenoid cell (arrow). (b) Coronal reformatted view through the sphenoid in bone reconstruction algorithm showing disruption of the floor of the right optic canal (arrow). Observe calcium deposits within the sphenoid sinus (dotted arrow) and bone thickening of the sphenoid walls.

**Figure 2:**
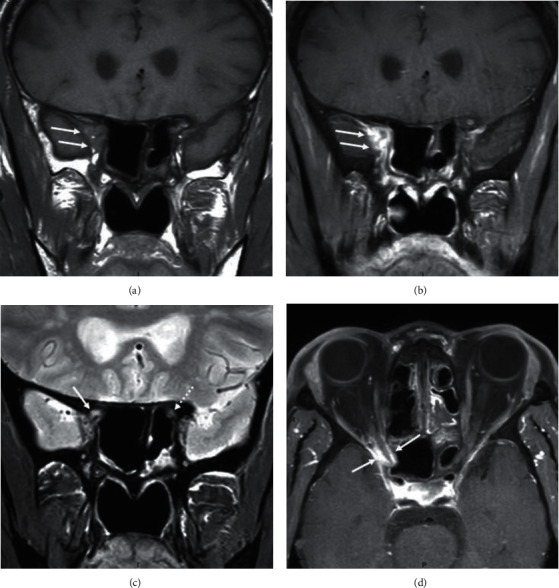
Orbital magnetic resonance imaging. (a) Coronal unenhanced T1-weighted view showing filling of the right sphenoid fissure by inflammatory material (double arrow). (b) Coronal contrast-enhanced T1-weighted view with fat suppression option in similar slice location as (a) showing intense enhancement of the inflammatory material (double arrow). (c) Coronal T2-weighted view showing abnormal hyper signal intensity of the intracanalar segment of the right optic nerve (arrow) when compared with normal contralateral side (dotted arrow). (d) Coronal contrast-enhanced T1-weighted view with fat suppression option showing a “tramtrack”-like encasement of the intracanalar segment of the right optic nerve (between arrows).

**Figure 3:**
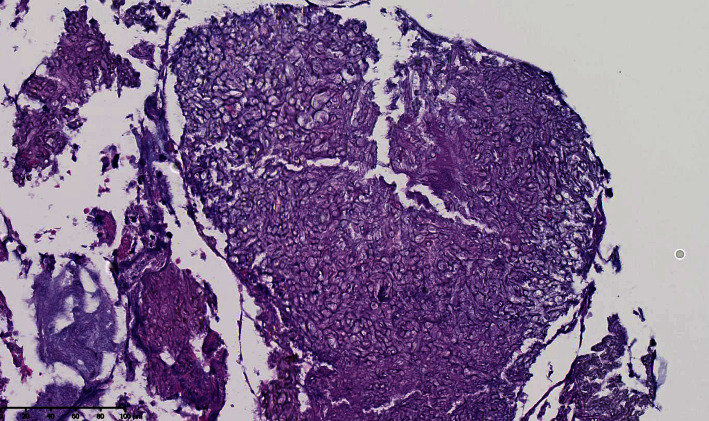
Histopathologic examination of the sinus material stained with periodic acid Schiff stain (PAS) showing fungal hyphae morphologically consistent with *Aspergillus*.

A 55-year-old man on hemodialysis for diabetic nephropathy received a kidney transplant from a living donor in March 2018. His past medical history was relevant for malaria, pulmonary tuberculosis, and chronic pancreatitis. His treatment included tacrolimus (8 mg q.d), methylprednisolone (4 mg q.d), mycophenolate mofetil (500 mg b.i.d), aspirin (100 mg q.d), pantoprazole (40 mg q.d), pancrelipase delayed-release capsule (20000 units t.i.d), and insulin. In June 2019, he was admitted for fatigue, photophobia, right hemicranial headache, and progressive vision loss of the right eye. Clinical examination revealed a normal left eye with normal vision (10/10). On the right side, there was a ptosis with unreactive pupil, a complete afferent pupillary defect and a total paralysis. The vision was “no perception of light,” and the slit lamp examination was within normal limits except for a moderate cataract which did not explain the complete vision loss and the unreactive pupil. The fundus did not reveal any optic nerve oedema or atrophy, and vessels occlusions were ruled out. A diabetic retinopathy with panretinal photocoagulations was described in both eyes. Intraocular tension was 14 mmHg in both eyes. Head computed tomography (CT) scanner demonstrated pansinusitis with diffuse mucosal thickening embedding punctate calcifications, chronic bone thickening, and focal osteolysis of the floor of the right optic canal (Figure 1). Orbital magnetic resonance imaging (MRI) confirmed the massive inflammatory extension to the sphenoid fissure and the encasement of the intracanal segment of the right optic nerve by the process (Figures 2(a)-2(c)). Intrinsic tissue damage to the optic nerve was present (Figure 2(d))No sign of intracranial extension was observed. Empirical antibacterial therapy with piperacillin-tazobactam and antifungal therapy with intravenous liposomal amphotericin B (LAmB) at the dosage of 3 mg/kg were empirically started. Surgical bilateral maxillectomy, ethmoidectomy, and sphenoidotomy were performed. Pathological examination of surgically resected specimens showed the presence of fungal hyphae (Figure 3). Voriconazole was therefore added (at the dosage of 6 mg/kg b.i.d. intravenously on day 1 followed by 4 mg/kg b.i.d. during 14 days and then oral 200 mg b.i.d). Microbiological culture of the surgical samples showed *Aspergillus flavus*, which was confirmed by polymerase chain reaction (PCR), consistent with a proven sino-orbital aspergillosis. LAmB and mycophenolate mofetil were stopped. Voriconazole was pursued, and dosage of tacrolimus was tapered to reach a trough level of 3 to 4 ng/ml. In the meantime, culture of the surgical samples showed coinfection with *Pseudomonas aeruginosa* and *Staphylococcus epidermidis*, which was treated by ceftazidime and cefazolin. The patient was discharged six weeks later, with improved general status and relief of pain. While the eye motility recovered completely, the vision did not. Follow-up MR examination nine months later showed regression of the pansinusitis together with a right optic atrophy. At one year, the lesions on MRI were stable without signs of active inflammation, thereby allowing discontinuation of voriconazole. Three months after interruption of antifungal treatment, the patient is still doing well without neurological complaints except for right blindness. As expected, the last fundus exam confirmed major right optic nerve pallor. The slit lamp examination remains unchanged. Kidney graft function remains stable as well as MR features.

## 3. Discussion

*Aspergillus* are ubiquitous saprophytic dichotomously branching fungi mainly present in hot, dry, and dusty climates. Infection occurs via inhalation of fungal spores [3]. Invasive aspergillosis (IA) is the most common fungal infection in solid organ transplant (SOT) recipients after candidiasis. It has been reported in 1 to 15% of SOT recipients and in 0.5 to 4% of KTR [1, 4–6]. IA most frequently affects lungs and sinuses. Orbital involvement is uncommon [7]. Only few case reports and small case series of *Aspergillus* optic neuritis have been reported up to now [1, 2, 7–12]. In most cases, orbital aspergillosis occurs mainly through the paranasal sinuses spreading by bony erosion or through vessel walls. Invasive aspergillosis can remain localized or become fulminant with multiple organ involvement [1, 8, 9].

Although it has improved over the past two decades, the diagnosis of *Aspergillus* infections remains difficult and is highly dependent on appropriate clinical suspicion [2, 10]. In 2019, an expert consensus group revised the definitions of invasive fungal disease [13]. The diagnosis of a proven invasive fungal disease requires the histologic demonstration of fungal elements within associated tissue damage, irrespectively of host factors or clinical features. More specifically, the diagnosis of a proven invasive aspergillosis as in our patient requires the identification of *Aspergillus* by culture or PCR in the affected tissue [13]. If the histological proof of fungal elements within tissue is lacking, the diagnosis of probable invasive fungal disease is made by the combination of host, clinical, and mycological evidences (Table 1) [13]. A possible invasive fungal disease is defined by the combination of host and clinical features without mycological support [13].

Symptoms of invasive aspergillosis depend on the affected organ. In orbital aspergillosis, the patient presents with unspecific initial symptoms such as persistent unilateral headache or retrobulbar pain, eye redness, and proptosis. Over time, symptoms may worsen to acute pain, cranial nerve palsies, amaurosis, and orbital apex syndrome [2, 11]. Symptoms of the eyes often precede those of the sinuses [11]. Orbital aspergillosis mimics other common optic neuritis. A key feature is the intensity of the pain described as unbearable. Misdiagnosing leads to inappropriate treatment and dire consequences [11].

Performing orientated neuroimaging is mandatory as illustrated by our patient. CT scanner is usually the most available imaging modality. Dense intraluminal calcifications in the sinuses are highly suggestive of fungal infection but their absence does not rule out the diagnosis. Bone erosions allowing sino-orbital way of spreading are commonly seen but at late stage of the disease course and not systematically because the agent may spread through vessel walls. The benefit of MRI relies in enhanced soft tissue contrast allowing better delineation of the extent of the inflammation towards orbits together with sensitive detection of tissue damage to the optic nerve [8, 9, 12].

Every effort should be made to obtain adequate amounts of tissues for microbiological and pathologic examination. Fine-needle aspiration or biopsy of the sinus or the orbit is required. As in our patient, pathological examination may show the characteristic dichotomously branching septate hyphae. The most helpful stains for visualizing fungi are the methenamine silver stain and the periodic acid-Schiff (PAS) stain [12]. Repeated biopsies are often necessary due to frequent inconclusive results. Antigen-based diagnosis relies on detection of either galactomannan or bêta-D-glucan, two constituents of fungal-cell walls. The first can be performed on plasma, serum, bronchoalveolar, or cerebrospinal fluid, while the latter is mainly limited to serum. The galactomannan test is more specific than beta-D-Glucan. A meta-analysis of studies about serum galactomannan test for early diagnosis of invasive aspergillosis in SOT recipients reported sensitivity and specificity of 22% and 84%, respectively (cutoff value not mentioned) [6]. Lately, the expert group recognized the usefulness of PCR methods for confirming the diagnosis of *Aspergillus* disease [13]. Moreover, PCR is able to detect both genus, species, and certain mutations associated with triazole resistance.

Currently, treatment of invasive sino-orbital aspergillosis is based on the guidelines established for the treatment of general invasive aspergillosis [1, 2, 12]. Former therapy included the use of systemic amphotericin B (deoxycholate), but this treatment has a number of toxicities (including renal dysfunction), and the mortality once intracranial spread has occurred is 80%. If fungal infection is initially suspected, broad empirical antifungal therapy like LAmB should be quickly started to cover other fungal infections like mucormycosis. After identification of *Aspergillus*, antifungal therapy should be switched to voriconazole. The latter is recommended as the first treatment for IA by the guidelines of the Infectious Diseases Society of America (IDSA), the American Thoracic Society (ATS), and the European Society for Clinical Microbiology and Infectious Diseases (ESCMID), after demonstrating a 22% survival benefit with better tolerance and lower toxicity over amphotericin B deoxycholate [14–17]. It is recommended as the drug of choice for treatment of invasive aspergillosis including in SOT recipients [6, 14]. Voriconazole trough level should be monitored [18]. Lately, isavuconazole has been shown as noninferior to voriconazole with fewer adverse events [19]. There is a paucity of data to guide the administration of antifungal therapy in patients with IA resistant or refractory to voriconazole: lipid amphotericin B formulation is recommended; posaconazole, itraconazole and echinocandins may be other alternatives [1–3, 6, 17]. While in immunocompetent hosts the recommended duration of therapy is 6 to 12 weeks minimum, it should be prolonged in immunocompromised hosts and depends on the clinical evolution. Aggressive surgical debridement of the affected sinonasal and orbit tissue with clean margins is strongly recommended but is often complicated by difficulty in determining the precise extent of the lesion and needs to be considered on an individual basis [1, 11, 12, 17].

Special aspects must be considered in the context of transplantation. Drug interactions between antifungal agents and immunosuppressive drugs must be carefully evaluated. The triazole agents are potent inhibitors of the CYP3A4 isoenzymes and have the potential to increase the levels of calcineurin-inhibitor agents (CNI) and sirolimus. A 50-60% reduction in the dose of CNI agents may be necessary with the concurrent use of voriconazole [6]. Also, reducing global immune suppression is recommended but can be challenging [14, 15]. For example, conflicting results exists about the effect of glucocorticoid use among SOT patients [20].

The prognosis of invasive sino-orbital aspergillosis remains poor. Despite appropriate treatment, many patients definitely lose vision and even die few days or months after the initial symptoms [9, 11]. In overall cases of invasive aspergillosis, the mortality rate decreased to 25% since the use of voriconazole [6, 16]. Poor outcome may be attributed to delay in diagnosis, cerebral extension, and recurrence of *Aspergillus* within involved tissues, in spite of extensive debridement and long duration antifungal treatment, particularly in the immunocompromised hosts [11].

In conclusion, sino-orbital invasive aspergillosis is an uncommon fungal infection mostly seen in immunocompromised hosts who present with acute and painful visual loss. A high index of suspicion is required for adequate diagnosis. Pathological and microbiological analysis of multiple surgical samples may be necessary for diagnosis and prompt start of appropriate therapy. Surgical debridement and prolonged course of antifungal therapy as well as reduction of immunosuppression are currently the cornerstones of IA management. Overall outcome is poor and depends on the host factors and the extension of aspergillosis.

## Figures and Tables

**Table 1:** Host factors, clinical, and mycological criteria for the diagnosis of probable sino-orbital fungal infection in solid organ transplant recipient (adapted from ^11^).

Host factors	Receipt of a solid organ transplant
Clinical features	Acute localized pain (including pain radiating to the eye)
	Or nasal ulcer with black eschar
	Or extension form the paranasal sinus across bony barriers, including into the orbit

Mycological evidence	Mold recovered by culture of sinus aspirate samples or microscopic detection of fungal elements in sinus aspirate samples indicating a mold
	Or for *Aspergillus*: galactomannan antigen detected in plasma, serum, or CSF
	Or for *Aspergillus*: ≥2 PCR tests positive in plasma/serum/whole blood

## Data Availability

The datasets used during the current study are available from the corresponding author on reasonable request.
